# The Effects of Unilateral Labyrinthectomy on Monoamine Neurotransmitters in the Medial Vestibular Nucleus of Rats

**DOI:** 10.3390/biom13111637

**Published:** 2023-11-10

**Authors:** Jun Wang, E Tian, Yuejin Zhang, Zhaoqi Guo, Jingyu Chen, Weijia Kong, Yisheng Lu, Sulin Zhang

**Affiliations:** 1 Department of Otorhinolaryngology, Union Hospital, Tongji Medical College, Huazhong University of Science and Technology, Wuhan 430022, China; ent_wangjun@hust.edu.cn (J.W.); etian@hust.edu.cn (E.T.); d202381955@hust.edu.cn (Z.G.); m202175891@hust.edu.cn (J.C.); entwjkong@hust.edu.cn (W.K.); 2 Institute of Otorhinolaryngology, Union Hospital, Tongji Medical College, Huazhong University of Science and Technology, Wuhan 430022, China; 3 Department of Physiology, School of Basic Medicine, Huazhong University of Science and Technology, Wuhan 430030, China; yjzhang0621@163.com; 4 Institute of Brain Research, Collaborative Innovation Center for Brain Science, Huazhong University of Science and Technology, Wuhan 430030, China

**Keywords:** unilateral labyrinthectomy, high-performance liquid chromatography, monoamine neurotransmitters, vestibular compensation, medial vestibular nucleus

## Abstract

Background: This study aimed to investigate the effects of unilateral labyrinthectomy (UL) on monoamine neurotransmitters in the medial vestibular nucleus (MVN) of rats. Methods: Adult Sprague–Dawley rats were utilized for the vestibular impaired animal model through UL. The success of the model establishment and the recovery process were evaluated using vestibular behavioral tests, including spontaneous nystagmus, postural asymmetry, and balance beam test. Additionally, the expression levels of c-Fos protein in the MVN were assessed by immunofluorescence. Furthermore, changes in the expression levels of monoamine neurotransmitters, including 5-hydroxytryptamine (5-HT), norepinephrine (NE), dopamine (DA), and histamine in the MVN, were analyzed using high-performance liquid chromatography (HPLC) at different time points after UL (4 h, 8 h, 1 day, 2 days, 4 days, and 7 days). Results: Compared to the sham control group, the UL group exhibited the most pronounced vestibular impairment symptoms at 4 h post-UL, which significantly decreased at 4 days and almost fully recovered by 7 days. Immunofluorescence results showed a notable upregulation of c-Fos expression in the MVN subsequent to the UL-4 h, serving as a reliable indicator of heightened neuronal activity. In comparison with the sham group, HPLC analysis showed that the levels of 5-HT and NE in the ipsilesional MVN of the UL group were significantly elevated within 4 days after UL, and peaked on 1 day and 2 days, respectively. DA showed an increasing trend at different time points up to 7 days post-UL, while histamine levels significantly increased only at 1 day post-UL. Conclusions: UL-induced dynamic changes in monoamine neurotransmitters during the early compensation period in the rat MVN may be associated with the regulation of the central vestibular compensation mechanism by the MVN.

## 1. Introduction

Acute unilateral vestibular dysfunction represents the most severe manifestation within the vestibular system [1]. When vestibular sensory inputs are suddenly altered, an acute vestibular syndrome arises that includes severe rotational vertigo, postural imbalance at rest and during movement, spontaneous nystagmus, and oscillopsia [1,2]. Patients experience acute vestibular syndrome in cases of unilateral peripheral vestibulopathy, such as in vestibular neuritis, Ménière’s disease, or labyrinthine fistula [3]. The aforementioned disorders occur due to changes in the vestibulo-spinal and vestibulo-oculomotor reflexes, as well as modifications in vestibulo-cortical signals [2]. With the help of vestibular compensation, these imbalance symptoms typically resolve within two weeks, but they can cause significant discomfort during the recovery process [3,4]. Therefore, investigating the involvement of molecular mechanisms of vestibular compensation would have clinical benefits.

Currently, unilateral labyrinthectomy (UL) is widely performed in animal models of vestibular dysfunction [5]. The UL-induced asymmetry between bilateral vestibular nuclear activities produces severe oculomotor and postural symptoms [6]. However, the asymmetry disappears over time with the development of vestibular compensation in the brain [6,7]. During vestibular compensation, there is a gradual restoration of resting activity in the ipsilesional medial vestibular nucleus (MVN) neurons, and a ‘re-balancing’ of the resting firing rates on the two sides, approximately in parallel with the behavioral recovery [8]. The MVN plays a crucial role in processing and integrating vestibular inputs from the peripheral organs and modulating neurotransmitter release during vestibular compensation.

Monoamine neurotransmitters, such as 5-hydroxytryptamine (5-HT), norepinephrine (NE), dopamine (DA), and histamine, are essential signaling molecules in the central nervous system [9,10]. They regulate various physiological processes, including motor control [11], mood [12], and cognition [13]. Within the vestibular system, these neurotransmitters have been implicated in modulating the responses and plasticity of vestibular neurons [14,15]. Thus, understanding the impact of UL on monoamine neurotransmitters in the MVN is crucial for elucidating the neurochemical mechanisms underlying vestibular dysfunction, potentially helping to shorten the duration of acute vestibular disturbance symptoms.

In this study, we established an animal model of acute vestibular dysfunction by UL. We then employed high-performance liquid chromatography (HPLC) analysis to quantify the levels of monoamine neurotransmitters in the MVN at various time points after UL. Our findings revealed significant alterations in the levels of 5-HT, NE, DA, and histamine, particularly emphasizing the correlation between the dynamic alterations of multiple monoamine neurotransmitters and the recovery of vestibular behavior in rats. This knowledge may enhance our understanding of the neural mechanisms involved in vestibular compensation and have implications for the development of therapeutic interventions for vestibular disorders.

## 2. Materials and Methods

### 2.1. Animals

The experiments were performed on adult male Sprague–Dawley (SD) rats (200–240 g) purchased from the Experimental Animal Research Center of Hubei Province (Wuhan, China). All the in vivo surgery procedures were performed in accordance with the animal protocols approved by the Animal Welfare Committee of Huazhong University of Science and Technology. The rats were housed by three animals per cage in a temperature- and humidity-controlled room with a 12 h light/dark cycle, with food and water provided ad libitum. All efforts were made to minimize suffering and the number of animals used.

### 2.2. Experimental Designs

**Figure 1:**
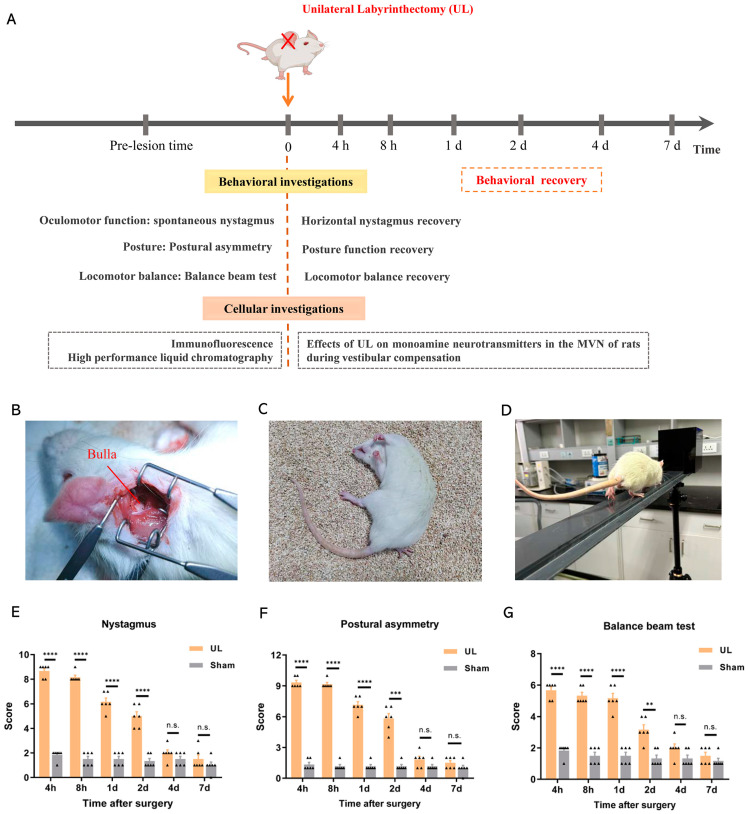
Establishment of the unilateral acute vestibular dysfunction model in rats. (**A**) Assessment of vestibular behavioral symptoms in rats at 4 h, 8 h, 1 day, 2 days, 4 days, and 7 days after UL, including spontaneous nystagmus, postural asymmetry, and balance beam test. MVNs were collected at different time points after UL for immunofluorescence and high-performance liquid chromatography analysis. (**B**) Illustration of UL in rats, exposing the bulla, indicated by the red line. (**C**,**F**) Postural asymmetry for evaluating postural and locomotor changes in rats, *n* = 6/group (F (3.06, 30.56) = 102.2, *p* < 0.0001). (**E**) Spontaneous nystagmus for evaluating eye movement function after model induction, *n* = 6/group (F (3.18, 31.76) = 72.17, *p* < 0.0001). (**D**,**G**) Balance beam test for evaluating the balance function in rats, *n* = 6/group (F (3.80, 38.02) = 38.08, *p* < 0.0001). Two-way analysis of variance (ANOVA) followed by Bonferroni’s post hoc test was used for comparisons between the UL and sham-operated groups. Data are presented as mean ± standard error of the mean (SEM). ** *p* < 0.01, *** *p* < 0.001, **** *p* < 0.0001, n.s. indicates no statistical difference. The black triangle in the figures (**E**–**G**) represent the behavioral score of each rat. UL: unilateral labyrinthectomy; MVN: medial vestibular nucleus.

We employed 42 SD rats in our experiments, achieving a modeling success rate of over 90%. (i) In the behavioral experiments, 12 SD rats were randomly divided into two groups, the UL (*n* = 6) and sham control (*n* = 6) groups. Behavioral assessments were conducted at different postoperative time points (4 h, 8 h, 1 day, 2 days, 4 days, and 7 days) (Figure 1A); (ii) in the immunofluorescence experiment, we selected another 6 SD rats that were randomly divided into two groups, the UL group (3 rats, with at least 3 slices per rat) and the sham control (3 rats, with at least 3 slices per rat) group. We focused on the time point of 4 h, representing the most severe vestibular injury symptoms after UL; (iii) for the HPLC analysis, we selected another 21 SD rats for surgery and randomly divided them into 7 subgroups based on different time points, with 3 rats in each subgroup.

### 2.3. Unilateral Labyrinthectomy (UL)

UL was performed following the previously described protocol to establish a model for vestibular compensation [16,17,18]. In brief, sodium pentobarbital (40 mg/kg) was used to induce anesthesia. A small incision was made behind the ear to expose the external ear canal and the tympanic bulla. Using an otologic drill, the lateral wall of the tympanic bulla was carefully opened (Figure 1B). Under microscopic guidance, the malleus and incus were gently removed while ensuring the preservation of the pterygopalatine artery. Subsequently, the oval window was opened and enlarged. The vestibule was aspirated using a fine plastic suction pipette, mechanically ablated to induce destruction, and then rinsed with 100% ethanol. To complete the procedure, the space resulting from the labyrinthectomy was filled with an absorbable gelatin sponge, and the skin wound was sutured. For the sham-operated control group, the same surgical procedure was performed without causing any damage to the inner ear.

### 2.4. Immunofluorescence

Rats were deeply anesthetized with sodium pentobarbital (65 mg/kg) and perfused transcardially with 100–150 mL normal saline, followed by 250–300 mL 4% paraformaldehyde (PFA) in 0.1 M phosphate buffer (pH = 7.4). The brain was gently removed, trimmed, and postfixed in the same fixative for 12 h at 4 °C. Subsequently, it was cryoprotected by immersion in a 30% sucrose solution for 48 h and frozen at 4 °C. Frozen coronal sections (30 um thick) containing the MVN were acquired by a freezing microtome (Thermo Scientific, HM550, USA). Brain sections were blocked in blocking buffer (10% goat serum and 3% BSA in PBS with 0.3% Triton X-100 included) for 1 h at room temperature, and then incubated in primary antibodies (rabbit anti-c-Fos: 1:500, ab208942, Abcam, Waltham, MA, USA) in the blocking buffer overnight at 4 °C. After washing with PBS 3 times, sections were incubated in secondary antibodies with the corresponding conjugated fluorophore (1:250, AS039, ABclonal, Wuhan, China) for 1 h at 37 °C. Images were taken using a confocal microscope (Zeiss, LSM800, Germany). The number of fluorescent cells was counted by ImageJ 1.53 t (National Institutes of Health, USA).

### 2.5. Sample Preparation and Ultra-High-Performance Liquid Chromatography

Rats were killed immediately after being deeply anesthetized, and their brain samples were promptly isolated and placed on a rat brain matrix immersed in ice-cold PBS. We performed coronal sections to extract the brainstem at two specific locations, 2 mm and 4 mm rostrally from the caudal margin of the fourth ventricle. The lateral border of the fourth ventricle and the brown-colored boundary between the prepositus hypoglossal nucleus and the dorsal paragigantocellular nucleus was employed as reference points for identifying the medial vestibular nuclei. Subsequently, we extracted a single tissue sample (1.8 mm in diameter) from each MVN in the caudo-rostral direction [19,20]. Finally, the MVN tissues were rapidly dissected out and kept at −80 °C until the time of the assay. MVN tissues (~30 mg) were extracted with 490 μL of pre-cooled methanol-water (2:1) using TissueLyser at 50 Hz for 90 s after mixing with 10 μL of the internal standards. The above extraction procedure was repeated twice. A total of 500 μL of the extraction was evaporated to dryness and re-dissolved in 50 μL of acetonitrile-water (1:4), and then aliquot (10 μL) was vortex-mixed with 87 μL borate buffer (0.2 M, pH 8.8) containing 20 mM TCEP and 5 mM ascorbic acid. After vortex mixing and standing for 30 s, 33 μL 5-AIQC solution was then added and incubated at 55 °C for 10 min. The mixture was cooled down to the ambient temperature and supplemented with 2 μL formic acid. The mixture was centrifuged at 12,000 rpm for 10 min at 4 °C, and the supernatant solution was filtered by a 0.22 µm membrane filter before ultra-high-performance liquid chromatography and electrospray ionization tandem mass spectrometry (HPLC-MS/MS) analysis.

The HPLC-MS/MS analysis was conducted using an Agilent 1290 HPLC coupled with an Agilent 6470 triple quadrupole mass spectrometer, which had an electrospray ionization (ESI) source (Agilent Technologies, USA). The 5-AIQC-tagged samples (1 μL each) were injected individually onto an HPLC column (Agilent ZORBAX RRHD Eclipse XDB C18 column, 2.1 × 100 mm, 1.8 μm particles) maintained at 50 °C. Two mobile phases, A and B, were used: A consisted of water with 0.004% formic acid and 5 mM ammonium bicarbonate, while B consisted of methanol with 0.16% formic acid and 2 mM ammonium formate. The flow rate was set at 0.5 mL/min. An optimized gradient elution was employed with the following scheme: 7–22% B (0–2 min), 22–30% B (2–5 min), 30–45% B (5–8.5 min), 45–95% B (8.5–8.6 min), and 95% B (8.5–12 min). Electrospray ionization was performed in the positive ion mode, with the nebulizer pressure set at 50 psi, the sheath gas temperature at 350 °C with a flow rate of 10 L/min, the dry gas temperature at 315 °C with a flow rate of 10 L/min, and the capillary set at 4000 V. Quantification of screening fragment ions was accomplished using multiple reaction monitoring techniques.

### 2.6. Behavioral Assessment

In behavioral tests, static symptoms of vestibular imbalance, including spontaneous nystagmus, posture asymmetry, and balance beam test. Behavioral testing was carried out by two experienced investigators who were blinded for the groups after surgery.

Spontaneous nystagmus scoring. The frequency of the fast-phase pullback of the cornea-retinal potential was used to evaluate spontaneous nystagmus [16,21]. The intensity of nystagmus was scored with 6–10 points, with 1 point for every 60 beats per minute (bpm). If spontaneous nystagmus was absent at rest, the animal was touched slightly. If this evoked nystagmus, a score of 1–5 points were given, with 1 point for every 60 bpm.

Postural asymmetry scoring. Postural deficits were scored as follows [22]: spontaneous barrel rolling, 10 points; barrel rolling that occurred after a light touch or blowing, 9 points; taking a lying position without any leg support toward the side of the lesion, 8 points; taking a lying position with leg support toward the side of the lesion, 7 points; turning in one direction or taking a lying position using the legs on the lesion side, 6 points; moving by using both legs, 5 points; wandering around while the head was rarely falling on the lesion side, 4 points; wandering around while the head was leaning on the lesion side, 3 points; if the asymmetry was difficult to detect, 2 points were given; and if the postural asymmetry was detectable only when the rat was lifted, 1 point was given.

Balance beam test. Motor coordination and balance were measured by the ability of the animals to traverse a horizontal balance beam of 150 cm in length and a diameter of 4 cm. A plastic platform was placed at one end of the rod as the start, and a black plastic box at the other end of the rod to motivate the animal in crossing the beam. Rats underwent a training session 3 days before the UL or sham procedure. To reduce stress and fatigue, the animals were allowed a 90 s rest between trials. The rats were trained to balance for 60 s on a short wooden beam raised 1 m off the floor. Once the rats were able to remain on the beam, they were evaluated for three consecutive trials per session and rated using a 6-point scale. The balance beam test was scored as follows [23]: 1 point; balances with steady posture (grooms, climbs barrier), 2 points; balances with unsteady posture (grasps sides of beam and/or has shaky movements), 3 points; hugs the beam or slips or spins on the beam, 4 points; attempts to balance, but falls off after 10 s, 5 points; drapes over or hangs from the beam, falls off in less than 10 s, 6 points; falls off, making no attempt to balance or hang onto the beam.

### 2.7. Statistical Analysis

Statistical analysis of experimental data was performed using GraphPad Prism 9.0 software (GraphPad Software Inc., San Diego, CA, USA). All data are presented as mean ± standard error of the mean (SEM). Multiple group comparisons at different time points were analyzed using one-way or two-way analysis of variance (ANOVA) followed by Bonferroni’s post hoc test. Comparisons between two groups were conducted using two-tailed Student’s *t*-test. A *p*-value less than 0.05 was considered statistically significant.

## 3. Results

### 3.1. Qualitative Evaluation of the Acute Vestibular Syndrome

After UL, the rats showed posture-locomotor alteration characteristics of the vestibular syndrome including spontaneous nystagmus, postural instability (Figure 1C), and vestibular imbalance (Figure 1D). These behavioral deficits were assessed using a qualitative “vestibular score” scale. Compared to the sham control group, the UL group exhibited a peak average vestibular score at 4 h and reached a plateau around the 4th day. By the 7th day, the vestibular function had mostly returned to normal levels (Figure 1E–G). There were no vestibular dysfunction symptoms identified from any time point of all the sham groups after the surgery.

### 3.2. The Excitation Marked by c-Fos Induction Is Enhanced in the Ipsilesional MVN after Unilateral Labyrinthectomy

**Figure 2:**
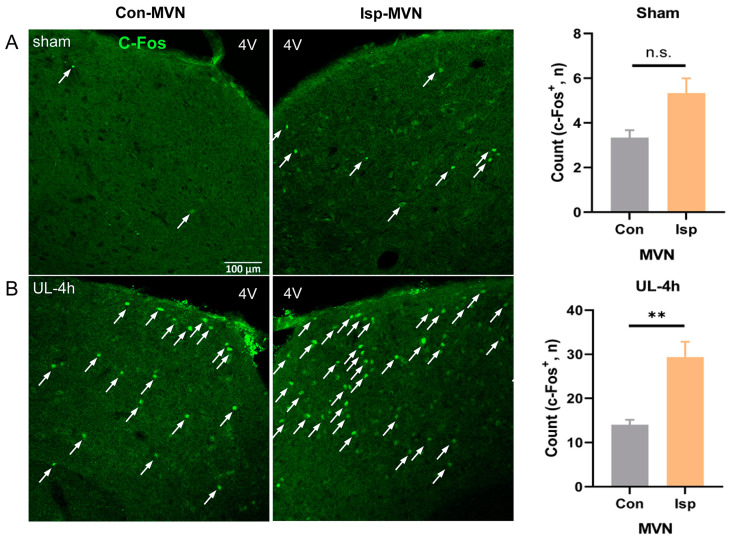
Massive activation of MVN neurons at 4 h after UL. (**A**) In the sham-operated group, a small number of c-Fos-positive neurons were activated in the ipsilesional medial vestibular nucleus (Isp-MVN) compared to the contralesional MVN (Con-MVN), n = 3/group, two-tailed Student’s *t*-test, *t* = 2.68, *p* = 0.055. (**B**) In the UL group, a significant increase in c-Fos-positive neurons was observed in both sides of the MVN, with the ips-MVN showing the most significant activation at 4 h after UL, n = 3, two-tailed Student’s *t*-test, *t* = 4.13. Arrows in white indicate c-Fos-positive neurons. Data are presented as mean ± SEM. ** *p* < 0.01, n.s. indicates no statistical difference. UL: unilateral labyrinthectomy; MVN: medial vestibular nucleus; 4V: fourth ventricle. Scale bar, 100 μm.

C-Fos is an early gene expression product commonly used as a marker of neuronal activity. In the sham-operated group, a small number of c-Fos-positive neurons were activated in the ipsilesional MVN compared to the contralesional MVN (*n* = 3, two-tailed Student’s *t*-test, *t* = 2.68, *p* = 0.055) (Figure 2A). In the UL group, a significant increase in c-Fos-positive neurons was observed in both sides of the MVN, with the ipsilesional side showing the most significant activation at 4 h after UL (*n* = 3, two-tailed Student’s *t*-test, *t* = 4.13, *p* = 0.015) (Figure 2B). Therefore, the pronounced increase in c-Fos expression in the ipsilesional MVN following UL suggests enhanced neuronal activity in this region.

### 3.3. HPLC Analysis Reveals Elevated Levels of Monoamine Neurotransmitters during Vestibular Compensation

**Figure 3:**
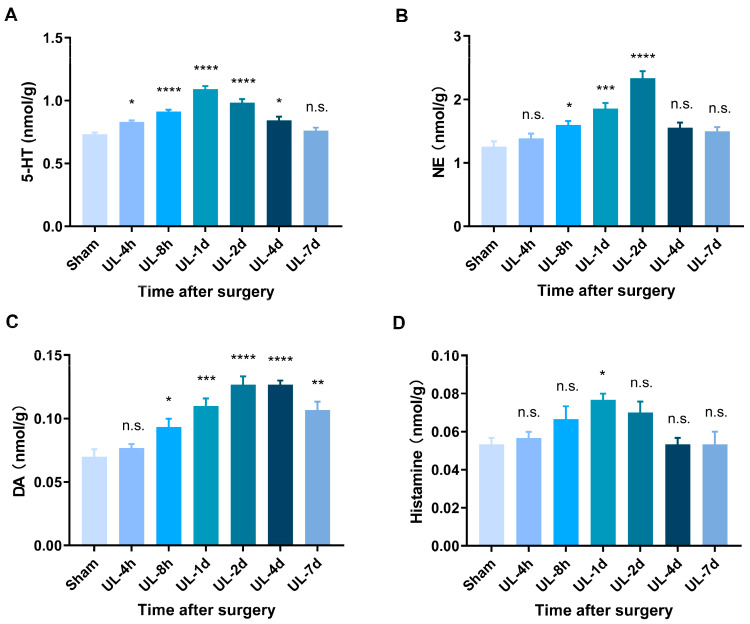
HLPC analysis reveals elevated levels of monoamine neurotransmitters after UL. (**A**) Levels of 5-HT significantly increased at 4 h, 8 h, 1 day, 2 days, and 4 days after UL, peaking at 1 day postoperatively and gradually decreasing around 4 days after UL, returning to preoperative levels by 7 days, *n* = 3/group (F (6, 14) = 32.39, *p* < 0.0001). (**B**) NE levels significantly increased at 8 h, 1 day, and 2 days after UL, peaking at 2 days postoperatively, and gradually decreasing around 4 days after surgery, returning to preoperative levels by 7 days, *n* = 3/group (F (6, 14) = 18.74, *p* < 0.0001). (**C**) DA levels significantly increased at 8 h, 1 day, 2 days, 4 days, and 7 days after UL, peaking at 2 days postoperatively, and gradually decreasing around 7 days after surgery, *n* = 3/group (F (6, 14) = 15.97, *p* < 0.0001). (**D**) Histamine levels significantly increased at 1 day after UL and gradually decreased to preoperative levels around 2 days after surgery, *n* = 3/group (F (6, 14) = 3.87, *p* = 0.017). One-way analysis of variance (ANOVA) followed by Bonferroni’s post hoc test was used for comparisons between the UL and sham-operated groups. Data are presented as mean ± SEM. * *p* < 0.05, ** *p* < 0.01, *** *p* < 0.001, **** *p* < 0.0001, n.s. indicates no statistical difference. HLPC: High-performance liquid chromatography; UL: unilateral labyrinthectomy; 5-HT: 5-hydroxytryptamine (serotonin); NE, norepinephrine; DA, dopamine.

In this study, we employed HPLC analysis to investigate the dynamic changes of neurotransmitters in the rat MVN following UL. It is interesting that the consistency between the changes in multiple monoamine neurotransmitters with the behavioral recovery of rats after UL. Our results showed that 5-HT and NE basal levels of MVN were high (0.733 ± 0.012 and 1.257 ± 0.081 nmol/g protein, respectively) (Table 1). Compared with the sham control group, the levels of 5-HT and NE in the ipsilesional MVN of the UL group were significantly elevated within 4 days after UL, and peaked on 1 day and 2 days, respectively (Figure 3A,B). Similarly, the levels of DA showed an increasing trend at different time points up to 7 days post-UL (Figure 3C), while histamine levels significantly increased only at 1 day post-UL (Figure 3D). 

## 4. Discussion

This study investigated the dynamic changes in monoamine neurotransmitters within the MVN at various time intervals following the UL using HPLC. Our research endeavors encompassed several key aspects. Firstly, we effectively established an animal model to mimic acute vestibular syndrome. Secondly, we observed a notable upregulation of c-Fos expression in the MVN at 4 h after UL, serving as a reliable indicator of heightened neuronal activity and endorsing the validity of our modeling strategy. Lastly, our findings indicated that UL dynamic alterations in monoaminergic neurotransmitter levels within the MVN during the process of vestibular compensation.

The process of vestibular compensation following unilateral vestibular loss is an intricate phenomenon that encompasses widespread synaptic, neuronal, and circuit plasticity mechanisms [24,25,26]. After UL in rats, we observed a significant increase in c-Fos activation within the ipsilesional MVN compared to the contralesional MVN at an early stage of vestibular compensation. This finding suggests an imbalance in neural activity between the two sides of the vestibular system following the surgical procedure. The excessive activation of c-Fos in the ipsilesional MVN indicates heightened neural response and increased metabolic activity in this region [27,28]. This hyperactivity may be a compensatory mechanism aimed at restoring the disrupted vestibular function caused by UL.

Vestibular compensation is a process in which the central nervous system adapts to the functional changes caused by a vestibular lesion or dysfunction [29]. During this compensation process, multiple neurochemical changes occur to restore balance and spatial orientation. In this study, HPLC analysis showed that the levels of 5-HT and NE in the ipsilesional MVN of the UL group were significantly elevated within 4 days after UL, and peaked on 1 day and 2 days, respectively. DA showed an increasing trend at different time points up to 7 days post-UL, while histamine levels significantly increased only at 1 day post-UL. While the association between vestibular dysfunction and changes in monoamine levels has been reported by previous studies, no consensus has been reached so far. Cransac et al. [19] investigated the effects of UL-6 h on the concentrations of monoamines (NE, DA, 5-HT) and their respective metabolites in albino and pigmented rats. They found the activation of NE and DA systems in albino rats, whereas no such changes were observed in pigmented rats. Interestingly, no 5-HT and 5-hydroxyindoleacetic acid (5-HIAA) changes were found in either brainstem nuclei of albino rats [19]. However, Zhai et al. [30] explored the concentration alterations of monoamine neurotransmitters in balance/anxiety-related nuclei of intratympanic gentamicin (GT)-induced balance disorder, they found three days after GT administration, the concentration of NE and 5-HIAA within MVN increased significantly compared with the control group. The aforementioned discrepancies among studies might be due to the different timing of tissue sampling and vestibular functional injury models.

Our findings revealed significant alterations in the levels of 5-HT, NE, DA, and histamine, particularly highlighting the similarities between the changes in multiple monoamine neurotransmitters with the behavioral recovery of rats. Regarding 5-HT, we observed a marked increase in its concentration immediately after UL, followed by a gradual decline over time. Previous research has provided evidence supporting the involvement of 5-HT in the modulation of the vestibular-ocular reflex, which affects eye movement in awake and sleeping rats [9,31,32]. These findings strongly suggest that 5-HT plays a crucial role in the regulation of vestibular circuits responsible for maintaining posture. Similarly, NE exhibited a time-dependent response, with a notable elevation in its levels during the acute phase post-UL. This surge in NE could be attributed to the activation of the sympathetic nervous system in response to the vestibular insult. As the recovery process ensues, NE levels gradually return to baseline, suggesting the involvement of noradrenergic modulation in vestibular compensation [33,34]. Moreover, the observed elevation of DA levels in the MVN of rats further supports the involvement of this neurotransmitter. Previous studies have indicated that dopaminergic agonists facilitate the compensation of postural and ocular symptoms while reducing the clinical manifestations induced by UL in rats [24,35]. Additionally, our findings demonstrated a significant temporal variation characterized by an initial increase in histamine levels, followed by subsequent recovery. Histamine, known for its role in modulating arousal and attention, may play a crucial role in facilitating the adaptive plasticity of vestibular circuits [36,37]. These changes in histamine levels may reflect an adaptive response aimed at restoring homeostasis and facilitating the restoration of vestibular function [38,39].

Our study has its own strengths. We conducted the first comprehensive assessment of dynamic changes in monoamine neurotransmitters following UL in rats at different time points within the first week. Previous studies have primarily focused on investigating changes in monoamine neurotransmitters immediately after UL, limited to a few hours. The correlation between the temporal patterns of monoamine neurotransmitters and behavioral recovery provides valuable insights into the neurochemical processes underlying vestibular compensation. By linking changes in monoamine neurotransmitter levels with specific behavioral milestones, we can potentially identify key neurotransmitter systems and their temporal dynamics associated with the recovery process.

Our study also suffers from some limitations. Firstly, this study acknowledges the limitation of a small sample size in UL of rats, warranting future investigations for validation and broader generalization. Nonetheless, the selected animals underwent rigorous screening and met stringent inclusion criteria to ensure the validity and reliability of the results. Secondly, due to the involvement of multiple time points after UL, our focus was limited to analyzing the changes in monoamine neurotransmitters within the ipsilesional MVN. The contralesional MVN and other brain regions, such as the locus coeruleus, were not further examined. Thirdly, the sham control group was not analyzed longitudinally by HLPC from different postoperative time points, but only at baseline. However, our immunofluorescence staining result did not observe any c-Fos difference in the sham group MVN with surgical procedure, suggesting the neural activity is normal 4 h after surgical procedure. It is essential to overcome this limitation in future research to explore neurotransmitter changes in these areas, providing a more comprehensive understanding of the effects of UL.

## 5. Conclusions

In conclusion, our study expands upon previous research by assessing the temporal changes in monoamine neurotransmitters following UL over the course of the first week, aligning them with the behavioral recovery of rats. This research provides a more realistic and meaningful perspective on the neurochemical dynamics associated with vestibular compensation and opens new avenues for future research and clinical applications.

## Figures and Tables

**Table 1:** The effect of unilateral labyrinthectomy on monoamine neurotransmitters in the medial vestibular nucleus of rats.

Group	5-HT	NE	DA	Histamine
Sham	0.733 ± 0.012	1.257 ± 0.081	0.070 ± 0.006	0.053 ± 0.003
UL-4 h	0.830 ± 0.012	1.387 ± 0.074	0.077 ± 0.003	0.057 ± 0.003
UL-8 h	0.913 ± 0.015	1.600 ± 0.061	0.093 ± 0.007	0.067 ± 0.007
UL-1 d	1.090 ± 0.025	1.857 ± 0.088	0.110 ± 0.006	0.077 ± 0.003
UL-2 d	0.983 ± 0.030	2.333 ± 0.114	0.127 ± 0.007	0.070 ± 0.006
UL-4 d	0.843 ± 0.028	1.553 ± 0.082	0.127 ± 0.003	0.053 ± 0.003
UL-7 d	0.760 ± 0.025	1.497 ± 0.068	0.107 ± 0.007	0.053 ± 0.007

Note: The values are expressed as nmol/g protein and the data presented as the mean ± SEM (*n* = 3/group). 5-HT, 5-hydroxytryptamine; NE, norepinephrine; DA, dopamine; UL: unilateral labyrinthectomy.

## Data Availability

Data are contained within the article.
